# Vital Sign Measurement and Response to Abnormal Measures in Surgical Specialty Clinics

**DOI:** 10.1001/jamanetworkopen.2022.9491

**Published:** 2022-04-26

**Authors:** Matthew Shneyderman, Edward Yin, Adam Levin, Oluseyi Aliu, Daniel Sun, Andrew J. Cohen

**Affiliations:** 1 Department of Krieger Arts and Sciences, Johns Hopkins University, Baltimore, Maryland; 2 Department of Orthopedic Surgery, The Johns Hopkins University School of Medicine, Baltimore, Maryland; 3 Department of Plastic and Reconstructive Surgery, Johns Hopkins Hospital, Baltimore, Maryland; 4 Department of Otolaryngology–Head and Neck Surgery, Johns Hopkins University School of Medicine, Baltimore, Maryland; 5 Trauma and Reconstructive Urologic Surgery, Brady Urological Institute, Johns Hopkins University, Baltimore, Maryland

## Abstract

This cohort study assesses patient presentation to subspeciality clinics and whether normal vital signs and abnormal vital signs are addressed.

## Introduction

Vital signs indicate the physiological condition of a patient, are associated with hospital readmissions, and help guide the level of care.^1,2^ A 2013 study^3^ found that vital signs are not regularly recorded and that vital sign measurement accuracy is low. There is a paucity of evidence regarding the utility of vital sign measurement in outpatient settings despite mandatory reporting policies. The evaluation of vital signs becomes futile when, for example, a patient presenting to a clinic has high vital sign values that are not noted, recognized, or acted on. We hypothesized that most patients presenting to subspeciality clinics have normal vital sign measurements and that abnormal vital sign measurements will not be addressed.

## Methods

In this cross-sectional study, we performed a retrospective medical record review of outpatients presenting to urology, plastic surgery, orthopedics, and otolaryngology specialists (convenience and homogenous sample given a priori similar practice patterns) from October 2019 to January 2020 at a tertiary care center in Baltimore, Maryland. The STROBE reporting guideline was followed. The Johns Hopkins institutional review board deemed this study to be exempt from review and approval because data were obtained from medical records. All vital signs entries (blood pressure, pulse rate, oxygen saturation measured by pulse oximetry, temperature, and respiratory rate) were collected. Hypertension was defined by a systolic blood pressure (SBP) measurement greater than 130 mm Hg or a diastolic blood pressure measurement (DBP) greater than 80 mm Hg, and hypotension was defined by an SBP less than 90 mm Hg or a DBP less than 60 mm Hg, per the American Heart Association guidelines.^4^ A pulse rate less than 60/min or greater than 100/min, a temperature less than 95 °F or greater than 99.6 °F, and a blood oxygen level of less than 95% defined abnormal levels. Demographic characteristics, day and time of the appointment, tardiness, visit result, Charlson Comorbidity Index, insurance, language, medications, vital signs available, and problem list were recorded. Categorical variables were compared using the χ^2^ tests, and continuous variables were measured using the Kruskal-Wallis test. The threshold for statistical significance was set at 2-sided *P* < .05. Data analysis was performed using Stata, version 17.0 (StataCorp).

## Results

Of 442 patients included, median age was 58 years (range, 10-93 years), 229 (51.8%) were male, and 267 (60.4%) were of White race. The median age of outpatients with normal vital sign measurements was lower than the median age of patients with abnormal vital sign measurements (Table 1). One hundred-forty six outpatients with abnormal vital sign measurements (49.8%) had private insurance and 105 outpatients with normal vital sign measurements (70.5%) had private insurance. At least 3 vital sign measurements were checked in 107 encounters (24.2%). No patient had all 5 vital signs checked. Three vital sign measurements were checked in 90% of visit for plastic surgery and 15% of visits for otolaryngology. At least 1 vital sign was abnormal for 293 patients (66.3%) (Table 1). Pulse rate and blood pressure level were most commonly abnormal and recorded (Table 2). One patient incurred an overtly erroneous temperature reading of 36 °F and another of 125 °F. A total of 2 of 442 patients (0.45%) were referred to the emergency department for BP of 162/104 mm Hg and 74/48 mm Hg, including 1 patient who developed preoperative syncope. Otherwise, there was no documentation in the medical records for any other patient with abnormal vital sign measurements to discuss with their primary care doctor, seek emergency departments care, or take additional medication.

**Table 1.** Characteristics of the Study Population

Characteristic	No. (%)			*P* value^a^
	Total (N = 442)	With abnormal vital sign measurements (n = 293)	With normal vital sign measurements (n = 149)	
Age, median (range), y	58 (10-93)	61 (13-92)	49 (10-93)	.01
BMI, median (range)	27.3 (20.1-57.3)	28.9 (20.1-57.3)	25.6 (19.1-83.1)	.37
Sex				
Female	213 (48.2)	128 (43.7)	85 (57.0)	.74
Male	229 (51.8)	145 (49.5)	84 (56.4)	
Race and ethnicity^b^				
African American	117 (26.47)	81 (27.6)	36 (24.2)	.23
Asian	27 (6.11)	12 (4.1)	15 (10.1)	
White	267 (60.41)	163 (55.6)	104 (69.8)	
Other	31 (7.01)	17 (5.8)	14 (9.4)	
Specialty visited				
Urology	203 (45.93)	131 (44.7)	72 (48.3)	.24
Plastic surgery	72 (16.29)	44 (15.0)	28 (18.8)	
Orthopedics	86 (19.46)	55 (18.8)	31 (20.8)	
Otolaryngology	81 (18.33)	43 (14.7)	38 (25.5)	
Smoking status				
Former	129 (29.19)	81 (27.6)	48 (32.2)	.72
Current	33 (7.47)	24 (8.2)	9 (6.0)	.13
Insurance				
Medicare	138 (31.22)	92 (31.4)	46 (30.9)	.02
Private	251 (56.79)	146 (49.8)	105 (70.5)	
International	10 (2.26)	7 (2.4)	3 (2.0)	
Self-pay	6 (1.4)	3 (1.0)	3 (0.7)	
Worker’s compensation	1 (0.2)	0 (0.0)	1 (0.6)	
Other/none	36 (8.1)	25 (8.5)	11 (7.4)	
Previously taking blood pressure medication	175 (39.6)	119 (40.6)	56 (37.6)	.001
Previously taking heart medication	113 (25.6)	79 (27.0)	34 (22.8)	.03
Late (≥5 min) to visit	62 (14.0)	37 (12.6)	25 (16.8)	.24
Patient language				
English	416 (94.1)	275 (93.9)	141 (94.6)	.19
Spanish	7 (1.6)	4 (1.4)	3 (2.0)	
Arabic	11 (2.5)	9 (3.1)	2 (1.3)	
Other	8 (1.8)	5 (1.7)	3 (2.0)	
Appointment day of week				
Monday	162 (36.7)	114 (38.9)	48 (32.2)	.16
Tuesday	92 (20.8)	51 (17.4)	41 (27.5)	
Wednesday	71 (16.1)	47 (16.0)	24 (16.1)	
Thursday	72 (16.3)	50 (17.1)	22 (14.8)	
Friday	45 (10.2)	31 (10.6)	14 (9.4)	
Month of appointment				
October	104 (23.5)	70 (23.9)	33 (22.1)	.15
November	187 (42.3)	119 (40.6)	24 (16.1)	
December	134 (30.3)	93 (31.7)	72 (48.3)	
January	17 (3.8)	11 (3.8)	20 (13.4)	
Care team				
Physician and resident	91 (20.6)	58 (19.8)	33 (22.1)	.77
Physician and scribe	70 (15.8)	46 (15.7)	24 (16.1)	
Physician only	228 (51.6)	156 (53.2)	72 (48.3)	
Physician and nurse practitioner	53 (12.0)	33 (11.3)	20 (13.4)	

Abbreviation: BMI, body mass index (calculated as weight in kilograms divided by height in meters squared).

^a^ *P* < .05 determined statistical significance.

^b^ Race and ethnicity categories were determined by medical record choices/patient self-report. Further breakdown is not available for categories comprising “other.”

**Table 2.** Statistical Summary of Abnormal Vital Sign Measurements

Abnormal vital sign^a^^,^^b^^,^^c^	No. (%) (N = 273)	Mean (SD)	Percentile			Lowest measurement	Highest measurement
			25	50	75		
Hypertensive SBP	212 (77.7)	143.41 (11.17)	136	141	149	130	185
Hypertensive DBP	157 (57.5)	87.07 (5.46)	83	86	90	80	105
Hypotensive SBP	5 (1.8)	80.2 (6.34)	76	77	86	74	88
Hypotensive DBP	30 (11.0)	52.43 (7.75)	49	55	56	19	59
Pulse <60/min	33 (12.1)	55.15 (3.38)	53	55	58	45	59
Pulse >100/min	40 (14.7)	109.0 (7.81)	103	106	113	101	133

Abbreviations: DBP, diastolic blood pressure; SBP, systolic blood pressure.

^a^ Hypertension was defined by an SBP measurement greater than 130 mm Hg or a DBP greater than 80 mm Hg, and hypotension was defined by an SBP less than 90 mm Hg or a DBP less than 60 mm Hg. A pulse rate less than 60/min or greater than 100/min, a temperature less than 95 °F or greater than 99.6 °F, and a blood oxygen level of less than 95% defined abnormal levels.

^b^ Temperature not included because both abnormal values were overtly erroneous.

^c^ Oxygen saturation not included because all values were within the normal range (95%-100%).

## Discussion

Results of this cross-sectional study suggest that a reevaluation of the utility of vital signs in subspecialty clinics may be warranted. Vital signs were not universally measured, and the results rarely affected the clinical encounter. Limitations include that oral communication between clinicians and patients regarding abnormal vital sign measurements may not have been captured by the medical record. High vital sign measurements may have been due to anxiety or stress about upcoming tests or surgical procedures; however, this supposition may reinforce questions regarding utility. In addition, these data may not represent the practice patterns in other subspecialty clinic settings. Routine vital sign measurement may need to be more carefully integrated into the clinical workflow and their merits completely reevaluated in subspecialty clinics.
